# Introduction to the New “Early Reports of Innovation” Section

**DOI:** 10.13023/jah.0201.03

**Published:** 2020-01-26

**Authors:** Erin Bouldin, Tim Marema

**Affiliations:** Appalachian State University; Center for Rural Strategies

**Keywords:** Appalachia, clinical practice, rural health, health organizations, health agencies

## Abstract

The *Journal of Appalachian Health* is introducing a new section this issue. While the journal is centralizing some of the best research and commentary on Appalachian health, the editorial team felt that practice-focused groups, organizations, and agencies may not be fully represented in the publication.

In this issue, the *Journal of Appalachian Health* is introducing a new section: Early Reports of Innovation. While the journal is centralizing some of the best research and commentary on Appalachian health, the editorial team felt that practice-focused groups, organizations, and agencies may not be fully represented in the publication.

Early Reports of Innovation was designed to create a mechanism by which promising practices from individuals and organizations working to promote health and well-being in Appalachia can be reported by, and included in, the journal without requiring a formal peer-review process.

In this issue, we describe the effort to develop a more trauma-informed and resilient community in Western North Carolina.^1^ What started as a small meeting among a committed group of community members representing a variety of youth-serving organizations has grown into the Watauga Compassionate Community Initiative (WCCI), an organization and interconnected network of people, agencies, and events. The group has seen rapid growth in its membership and has engaged people from education, law enforcement, public health, health care, and other sectors.

The Watauga Compassionate Community Initiative has supported data analyses and resource-map development and has contributed to new policies in schools and the justice system that consider trauma history and incorporate treatment throughout their processes. Given the increasingly loud calls to comprehensively address adverse childhood experiences (ACEs)^2^ and mitigate their impacts, we hope this example might inspire ideas in other communities to connect existing work or consider new efforts to address ACEs.

The article includes resources that might be helpful as communities begin this work. If you are involved with or aware of an innovative practice aimed at improving health or well-being in your own community and would like to share your history and lessons learned, please reach out to us. We are particularly interested in programs or policies that have been used with some success in Appalachia and are likely to work in other parts of the region. We will ask you to complete a short set of questions [add hyperlink] and will provide support in writing and editing the brief report.

We hope that publishing an Early Reports of Innovation will offer the opportunity to highlight excellent work happening in communities and to facilitate connections and new community-based networks. We look forward to working with you to share your story.
